# Editorial: New Professionalism and the Future of Work: Interdisciplinary Perspectives on Transformations in Business-Health Relationships

**DOI:** 10.3389/fpsyg.2019.02193

**Published:** 2019-09-24

**Authors:** Gabriele Giorgi, Nicola Mucci, Annamaria Di Fabio, Antonio Ariza-Montes

**Affiliations:** ^1^Department of Human Science, European University, Rome, Italy; ^2^Department of Experimental and Clinical Medicine, University of Florence, Florence, Italy; ^3^Department of Education, Languages, Intercultures, Literatures and Psychology - Psychology Section, University of Florence, Florence, Italy; ^4^Department of Business Management, Universidad Loyola Andalucía, Seville, Spain

**Keywords:** future work skills, intrapreneurship, innovation, occupational health, business

## Introduction

This special issue provides new perspectives into the future of work and it focuses on how innovation, entrepreneurship, and the evolution of digital robotics could influence health and productivity of individuals and enterprises. The world of work is changing rapidly, especially in terms of increasing digitalization and robotic innovation. Such a scenario may represent an opportunity for workers who adapt themselves, but also a potential source of stress and poor well-being for those subjects less inclined to change (e.g., Salanova and Llorens, 2013; Berg-Beckhoff et al., 2017; Richardson, 2017; Leso et al., 2018).

The concept of the classic workplace is also deeply changing, due to the possibility of working anytime and everywhere using portable devices connected to the Internet. All these aspects have led to the development of new skills and the growth of opportunities for digital workers However, not all of the workforce is ready to face these changes. For these reasons, the complex relationships between a changing way of working and the consequent reactions of workers and companies, are worthy to be investigated with an empirical approach. Several scientific reports are aimed at deepening the understanding of these issues and suggesting proactive strategies to manage the complex and newsworthy challenges of the “future of work.”

The majority of the 11 manuscripts published in this special issue are empirical contributions, coming from different geographical regions, such as Eastern-Europe, South Europe, Africa, and India, and involving multiple research areas (organizational psychology, occupational medicine, management, technology, social sciences). These research areas offer a variety of perspectives on the consequences of an imbalance between the new job demands related to the future work and the coping strategies devised by workers. Moreover, these research areas contribute to the promotion of an interdisciplinary approach aimed at improving workers' general well-being, self-efficacy, satisfaction, and productivity.

The manuscripts, when considered together, bring out three relevant aspects. First, new technologies and digitalization have a significant impact on workers' performance and well-being. By virtue of the fact that both new skills and a great adaptability are required, not everyone is able to follow this epochal progress. Second, academic education and vocational trainings seem to be crucial to provide workers with high self-perceived employability, self-efficacy, and satisfaction. These training programs should be provided to both new generation and elderly workers, who are often less prone to change. Third, the existence of a good work climate, the promotion of a supportive work environment and the development of strong social relationships, can moderate the side effects of the future work on the overall workforce.

## Overview of Articles in this Research Topic

The 11 manuscripts published in the special issue investigated and discussed several crucial aspects of the future world of work.

Following the setting of this Research Topic, three manuscripts treat the consequences of new ways of organizing work, such as time-spatial flexibility, work-related smartphone use, and technological changes.

The use of portable devices constantly connected to the Internet is deeply changing the way work is performed, with putative effects on psychological well-being, work organization and performance. The article of Van Laethem et al., through a diary study conducted in a sample of 115 employees, investigated daily smartphone use after and during work and its association with psychological detachment and work engagement. Results suggest that an intensive smartphone use after work hampers employees' psychological detachment. Conversely, intensive smartphone use during work undermines their work engagement, but only when employees experience high workplace telepressure as well.

The study conducted by Wessels et al. considers the theoretical model of time-spatial job crafting, discussing its components and antecedents and explaining how time-spatial job crafting is related to positive work outcomes through a time/spatial-demands fit. Starting from some individual and organizational antecedents through the time-spatial job crafting, it could be possible to develop some positive outcomes at an individual and organizational level, such as work engagement, performance and work-life balance, personal job fit, and organizational commitment. The time-spatial flexibility seems to contribute to a better organization of work and social life, also influencing performance and well-being.

The paper by Ghislieri et al., through a short review, discussed two important open issues of the industry 4.0. In detail, they focused primarily on the relationship between workers and technological changes in the era of intense expansion of automation in the workplace, and how this can affect workers' well-being, employment and equality. Secondly, they pointed out how job transformation could influence knowledge and skills requirements in the work of the future. An interesting aspect is the crucial role of trainers, educators, and policymakers in preventing skill obsolescence and fostering the continuous development and update of the expertise required by the future of work.

Three articles are focused on the relationship between personal resources and individual outcomes.

In the first article, Dražić et al. investigated the relationship among self-perceived employability, ambition and locus of control, which is intended as where a person situates the causation of various life events. The study was conducted in a sample of undergraduate psychology students. The results show that career ambition plays a mediating role in the relationship between the locus of control and employability. Furthermore, students perceived personal capabilities and ambition as internal strengths and lack of ambition as a major internal weakness. In other terms, developing and sustaining career ambition could lead to students' perception of better employability, especially in some regions with high unemployment rates, and where the global economic crisis has been more intense.

In the second article, Atitsogbe et al. investigated, through a multi-group path analysis, the relationship between personal resources (in terms of career adaptability and general self-efficacy) and career outcomes (in terms of self-perceived employability and entrepreneurial intentions) in an overall sample of 550 subjects of a West African country. The results showed that career adaptability and general self-efficacy were positively related to self-perceived employability, while only general self-efficacy was related to entrepreneurial intentions. By engaging an activation of resources, career adaptability seems to be particularly relevant for employability. Considering the high unemployment rates in the region where the study was conducted, these results might provide insights into the occupational integration challenges in such contexts.

In the third article, Pedrazza et al. investigated job satisfaction and perceived self-efficacy within the context of residential child-care. They found that attachment style and length of service are antecedents of both work-related self-efficacy and job satisfaction. Moreover, the relational issues seem to play a role in shaping the educators' satisfaction at work.

Five studies explored the possible consequences of work climate on workers' outcomes.

The main objective of the study performed by Benevene et al. was to investigate how job satisfaction could mediate the relationship between physical and mental health, and happiness and self-esteem in a sample of 300 Indian teachers. Results of the multiple linear regression showed that job satisfaction fully mediates between both happiness and self-esteem, and health. These results suggest the importance of developing policies to promote job satisfaction among teachers, and the need to deepen the mechanisms of job dissatisfaction.

On the other hand, as reported in the study by Di Marco et al., a work climate characterized by a discriminatory environment can affect workers well-being, and this effect is partially mediated by job autonomy. Experiencing a discriminatory work environment can undermine workers' psychological well-being. Some job resources, such as job autonomy and social support, might reduce its negative effects. Anyhow, resources based interventions need to be tailored to workers' needs in order to obtain the best results.

The aim of the study conducted by Boštjančič et al. was to investigate the relationship between corporate volunteering programs and job characteristics, connected with work engagement. The results indicate that employees whose employers have implemented volunteering programs are more engaged and report higher levels of both autonomy and support from their co-workers and supervisors. As well as being more social responsible, companies that promote corporate volunteering climate could improve engagement and performance of workers.

Managing human resources to increase productivity and workers' outcomes is a main challenge for companies that want to excel in an increasingly competitive world of work. As reported in the article by Boštjančič and Slana, companies may use various approaches and activities to attract and develop talented employees in the so-called “war for talent.” With an exploratory approach and using the method of semi-structured interviews, the Authors collected information about 21 Slovenian professionals. They found that the majority of enrolled resources are annually evaluated in terms of achieved goals and provided performances. On average 7% of employees are recognized as talented, and the largest number of companies try to attract talented employees through various activities and the planned development of the employer's brand. Moreover, the majority of companies are transparent in their communications with talented employees, but the biggest challenge remains how to attract talented employees and how to position the organization as a desirable employer.

The study conducted by Van der Heijde et al. investigated the role of age in the relationship between perceptions of learning climate and self-rated and supervisor-rated employability in seven European countries (Germany, Greece, Italy, the Netherlands, Norway, Poland, and the United Kingdom). The results confirmed that the relationship between age and perceptions of learning climate is negative, the model also showed a strong positive relationship between learning climate and self-rated and supervisor-rated employability. Furthermore, perceptions of learning climate appeared important for employability irrespective of life or career stage This finding suggests the need to improve lifelong employability by means of a supportive and learning climate.

## Conclusions

Overall, the manuscripts included in this special issue reported findings from a cumulative sample of over 3,200 workers, and perspectives from 50 authors. This scenario suggest that the continuous transformations in the workplace represent a big challenge for both companies and workers. In particular, companies have a strong need to develop new strategies to improve both well-being and performance of workers, and to sustain employability of new generation of workers. On the other hand, elderly workers, who often have more difficulty managing new technologies, should be supported both with a specific learning climate and tailored training courses.

We believe that the changes in the world of work should be managed by offering adequate organizational strategies aimed to improve both satisfaction and well-being of workers, and to guide companies in the forthcoming “fourth industrial revolution.”

Considering the close link between human and organizational resources, simultaneously affected by the same work transformations, we would like to mention the *motto* of the Business@Health Laboratory of the European University of Rome (www.uerbusinesshealth.com): “*business doesn't exist without workers' health and workers' health is business.”* Our hope is that the manuscripts contained in this special issue can guide stakeholders in the improvement of organizational practices for all the professionals involved in this epochal changes, in the promotion of workers' well-being through a supportive work environment, in the design of increasingly effective training programs, and in the development of inclusive social and economic policies (e.g., Aronsson et al., 2017; Paganelli et al., 2018).

## Author Contributions

GG, NM, AD, and AA-M equally contributed to all the following issues of the Editorial: conception of the work, acquisition, analysis, or interpretation of data from the contributions, drafting the work and critically revising it, final approval of the version to be published, agreement to be accountable for all aspects of the work in ensuring that questions related to the accuracy or integrity of any part of the work are appropriately investigated and resolved.

### Conflict of Interest

The authors declare that the research was conducted in the absence of any commercial or financial relationships that could be construed as a potential conflict of interest.
